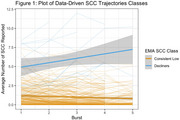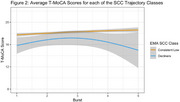# Longitudinal Increases in Subjective Cognitive Concerns are associated with Changes in Global Cognitive Performance: Findings from Daily Digital Diary Assessments in the Einstein Aging Study (EAS)

**DOI:** 10.1002/alz70863_110603

**Published:** 2025-12-23

**Authors:** Angel Garcia De La Garza, Carol A. Derby, Cuiling Wang, Nelson A. Roque, Mindy J. Katz, Richard B. Lipton, Laura A. Rabin

**Affiliations:** ^1^ Albert Einstein College of Medicine, Bronx, NY USA; ^2^ The Pennsylvania State University, University Park, PA USA; ^3^ Brooklyn College of the City University of New York, Brooklyn, NY USA; ^4^ The Graduate Center, CUNY, New York, NY USA

## Abstract

**Background:**

Ecological momentary assessment (EMA) is increasingly used to assess subjective cognitive concerns (SCCs), or self‐perceived memory and cognitive difficulties, without reliance on retrospective recall. While elevated SCCs are associated with increased risk of cognitive decline, little research has examined joint longitudinal trajectories of SCCs and cognitive function. We used smartphone‐based EMA to explore associations between SCC trajectories and trajectories in the telephone version of the Montreal Cognitive Assessment (T‐MoCA) scores in community‐dwelling older adults.

**Methods:**

Analyses included 219 Einstein Aging Study participants (mean age = 77.50, SD = 5.01; 69.86% female; 47.94% Non‐Hispanic White, 42.01% Non‐Hispanic Black, 10.05% Hispanic; 23.74% MCI; median follow‐up = 4 years, dementia‐free). Participants reported perceived cognitive lapses once daily at night over 14 days and repeated these assessments annually (2017–2022). Cognition was assessed via the validated 22‐item telephone Montreal Cognitive Assessment (T‐MoCA; normal cognition > 18). Latent class linear mixed‐effects models identified clusters of longitudinal changes in SCCs while adjusting for age, gender, race/ethnicity, cognitive status, and depression (GDS). Subsequently, we characterized T‐MoCA trajectories across these SCC groups using linear mixed‐effects models with piecewise splines to account for learning effects, adjusting for age, gender, and race/ethnicity.

**Results:**

We identified two SCC trajectory groups (Figure 1): (1) a consistently low SCC group (N = 199) with a low number of SCCs and a slight non‐significant longitudinal increase (SCC baseline mean = 0.74), and (2) a group with increasing SCC (N = 15) with a higher baseline mean mean (4.34) and a yearly increase of 0.81 SCCs (p < 0.001). These SCC trajectory groups exhibited distinct longitudinal patterns in the T‐MoCA (Figure 2). We found no group differences in T‐MoCA at baseline, nor did we observe significant differences in learning across the initial three annual assessments. The group with increasing SCCs showed declining T‐MoCA scores after the initial three annual assessments (p = 0.02), whereas the group with consistently low SCCs did not demonstrate decline.

**Conclusions:**

Longitudinal SCC trajectories correlate with distinct cognitive trajectories in the T‐MoCA. Our results indicate that increasing SCCs may predict declines in objective cognitive performance.